# Cognitive and Neural Correlates of Mathematical Giftedness in Adults and Children: A Review

**DOI:** 10.3389/fpsyg.2017.01646

**Published:** 2017-10-25

**Authors:** Timothy Myers, Emma Carey, Dénes Szűcs

**Affiliations:** Department of Psychology, Centre for Neuroscience in Education, University of Cambridge, Cambridge, United Kingdom

**Keywords:** math gifted, correlates of math giftedness, math expertise, math prodigy, high math performance, math achievement

## Abstract

Most mathematical cognition research has focused on understanding normal adult function and child development as well as mildly and moderately impaired mathematical skill, often labeled developmental dyscalculia and/or mathematical learning disability. In contrast, much less research is available on cognitive and neural correlates of gifted/excellent mathematical knowledge in adults and children. In order to facilitate further inquiry into this area, here we review 40 available studies, which examine the cognitive and neural basis of gifted mathematics. Studies associated a large number of cognitive factors with gifted mathematics, with spatial processing and working memory being the most frequently identified contributors. However, the current literature suffers from low statistical power, which most probably contributes to variability across findings. Other major shortcomings include failing to establish domain and stimulus specificity of findings, suggesting causation without sufficient evidence and the frequent use of invalid backward inference in neuro-imaging studies. Future studies must increase statistical power and neuro-imaging studies must rely on supporting behavioral data when interpreting findings. Studies should investigate the factors shown to correlate with math giftedness in a more specific manner and determine exactly how individual factors may contribute to gifted math ability.

## Introduction

A disproportionately large amount of scientific advancement throughout history has occurred due to cognitively gifted individuals. However, we know surprisingly little about the cognitive structure supporting gifted mathematics. The current understanding is that human mathematical ability builds on an extensive network of cognitive skills and mathematics-specific knowledge, which are supported by motivational factors (Ansari, 2008; Beilock, 2008; Fias et al., 2013; Szűcs et al., 2014; Szűcs, 2016). To date, most psychological and neuroscience studies have examined potentially important factors only in children and adults with normal mathematics as well as in children with poor mathematical abilities (e.g., in children with mathematical learning disability or developmental dyscalculia). In contrast, those with high levels of mathematical giftedness received relatively little attention. In order to facilitate research in this potentially important area, here we systematically review all studies aiming to uncover cognitive correlates of gifted mathematicians.

## Search strategy and criterion

We used two search strategies: (1) a computer search of databases, and (2) a review of reference lists of all articles retrieved. An electronic literature search was conducted, using the following database search engines without restriction on publication year: Google Scholar, Elsevier, PubMed, Scopus, and Web of Science. From the 4,652,956 total results, Titles were scanned for appropriateness through the first 20 pages of Google Scholar and all pages for the other search engines. Articles obviously having nothing to do with math giftedness were discarded. Search criteria (described below) were then applied to 15,800 articles, of which 62 were selected. For the complete list of search terms and the number of results per term, please see Appendix B in the supplementary materials.

Abstracts were reviewed by the first author and selected for further review if they met all of the following criteria: (1) written in English (2) had a participant or group of participants identified as math experts or math gifted. Articles were reviewed independently for relevance by two authors (TM and DS). The reference lists of selected articles were reviewed independently by both authors for further relevant articles.

## Review of the literature

The fragmented nature of evidence, that is, the use of very different tasks and the relatively low number of studies with similar deign and measures did not allow us to carry out a formal meta-analysis of effect sizes. Hence, we first discuss what factors individual studies identified as related to mathematical giftedness in adults and children. After this we draw some general conclusions.

### Information processing speed

Paz-Baruch et al. (2014) administered a battery of five speed of information processing tests to high school students (see Table 1; Row 1). The students were divided into four groups based on general and mathematical giftedness: (1) generally gifted and excellent in math (*n* = 41), (2) generally gifted and not excellent in math (*n* = 40), (3) not generally gifted and excellent in math (*n* = 53), (4) not generally gifted and not excellent in math (*n* = 56). The results showed that the generally gifted and excelling in math group outperformed the other three groups on all five tests. In the Crossing-out of Numbers and Simple Arithmetic tests, performance was associated with general intelligence and excellence in mathematics. With the other three tests, performance was only associated with general intelligence. Across the four groups, faster information processing speed was correlated with high mathematical ability only when general intelligence was also high (i.e., no effect of speed of information processing was seen with either condition of low general intelligence).

**Table 1:** Standardized tests and other measures used across studies.

**Row**	**Study**	**Tests**
1	Paz-Baruch et al., 2014	**Math Ability Measures:** Visual Matching and Crossing-out of Numbers subtests from the Woodcock-Johnson III (Woodcock et al., 2001), the Digit Symbol and Symbol Search subtest from the WISC-III (Wechsler, 1997), and timed simple arithmetic exercises (Openhaim-Bitton, 2003).
2	Kennedy and Walsh, 1965	**Cognitive IQ Measures:** Wechsler Adult Intelligence Scale (WAIS) (Wechsler, 1955), Stanford-Binet IQ, Kell-Hoeflin Incomplete Sentences (K-H), Allport-Vernon-Lindsey (AVL), Rotter Incomplete Sentences, Concept Mastery Test (CM), and the STEP assessment; **Personality Measure:** Minnesota Multiphasic Personality Inventory (MMPI.
3	Jensen, 1990	**Non-verbal Reasoning Measure:** Raven Advanced Progressive Matrices; **WM Measures:** forward and backward digit span subtests of the WAIS; **General Cognitive Ability Measures:** 3 elementary cognitive tasks.
4	Wu, 1996	**Environment Measures:** questionnaires and in-depth interviews.
5	Fehr et al., 2010	**Math Ability Measures:** four basic arithmetic operations with 1 or 2-digit numbers.
6	Fehr et al., 2011	**Calendrical Ability Measure:** calendar date task.
7	Prescott et al., 2010	**Spatial Processing Measure:** 3-dimensional object mental manipulation task.
8	Morsanyi et al., 2013	**Reasoning Measure:** transitive inference task (main task); **Math Ability Measures:** Mathematics Assessment for Learning and Teaching (MaLT; Williams, 2005), Numerical Operations subtests of the Wechsler Individual Achievement Test (WIAT-II; Wechsler, 2005); **Math Reasoning Measure:** Mathematical Reasoning subtest of the WIAT-II; **Verbal Ability Measures:** Hodder Group Reading Test-II (HGRT-II; Vincent and Crumpler, 2007), Word Reading subtest of the WIAT-II; **Verbal Reasoning Measure:** Psuedoword Decoding subtest of the WIAT-II; **Non-verbal Reasoning Measure:** Raven's Colored Progressive Matrices (Raven's CPM; Raven, 2008),; **General IQ Measure:** WISC-III).
9	Zhang et al., 2015b	**Verbal Reasoning Measure:** syllogism confirmation task.
10	Dark and Benbow, 1990	**Math Ability Measures:** equation writing task and algebra story problems, **WM Measures:** digit span, spatial span; **Verbal Ability Measures:** letter-digit continuous paired associates and letter-location continuous paired associates.
11	Dark and Benbow, 1991	**WM Measures:** digit span, letter span, word span, location span, and a continuous paired-association task utilizing digits, letters, and locations.
12	Dark and Benbow, 1994	**General Cognitive Measures**: simple categorization, dual-task recall, and continuous paired-association.
13	Pesenti et al., 2001	**Math Ability Measure:** arithmetic problem paired task (calculation and memory retrieval).
14	Tanaka et al., 2002	**Math Ability Measure:** delayed match-to-sample.
15	Swanson, 2006	**Math Ability Measures:** Mathematics subtest of the Wide Range Achievement Test (WRAT-III; Wilkinson, 1993) and the Mathematics subtest of the WIAT; **Reading Ability Measure:** WRAT-III reading, Rapid Digit Naming, and Rapid Letter Naming; **Non-verbal Reasoning Measure:** Ravens Advanced Progressive Matrices; **WM Measures:** WISC-III Forward Digit Span, Pseudoword Span, Word Span, WISC-III Backward Digit Span, Listening/Sentence Span, Digit/Sentence Span, Updating; **Visuo-spatial Processing Measures:** Visual Matrix task and Mapping task; **General Cognitive Measures:** Random Letter Generation, Random Number Generation, Categorical Fluency, Letter Fluency.
16	Leikin et al., 2013, 2014	**Non-verbal Reasoning Measure:** Ravens Advanced Progressive Matrices Test; **Math Ability Measure:** SAT-M; **WM Measures:** Digit Span and Working Memory for Digits and Letters subtests of the WISC-III, and a visuo-spatial WM task.
17	Ruthsatz et al., 2014	**General IQ and WM Measure:** Stanford-Binet IV & V (1986)
18	Zhang et al., 2015a	**Math Ability Measure:** arithmetic rule task with triangles and squares.
19	Barner et al., 2016	**General IQ Measure:** Woodcock-Johnson Tests of Achievement; **Math Ability Measures:** Math Fluency subtest of the WIAT and number comparison; **Non-verbal Reasoning Measure:** Raven's Progressive Matrices; **Visuo-spatial Processing Measure:** mental rotation task; **STM and WM Measures:** adaptive test of short-term memory and an adaptive test of visual WM.
20	Benbow and Minor, 1990	**Spatial Measures:** Guilford-Zimmerman Spatial Orientation test (Guilford and Zimmerman, 1981: ability to perceived arrangements of items of visual information in space); Guilford-Zimmerman Spatial Visualization test (visual transformations); Cubes (Benbow et al., 1983: form & manipulate mental images of objects); **Non-verbal Reasoning Measures:** Raven's Progressive Matrices, Advanced Set (Raven et al., 1977: apprehend relationships among meaningless figures and develop a systematic method of reasoning); **Mechanical Comprehension Measures:** Bennett Mechanical Comprehension Test, form AA (Bennett, 1940: understand various kinds of physical and mechanical relationships); **Vocabulary and General Knowledge Measures:** Terman's Concepts Mastery Test, Form T (Terman, 1956: process abstract ideas at an advanced level)
21	Robinson et al., 1996	**Quantitative Measures**-Stanford-Binet IV: Quantitative (Thorndike et al., 1986: miscellaneous word problems); Stanford-Binet IV: Number Series (predicting next two items in a series of numbers); Key Math, Revised: Numeration (Connolly, 1988: enumeration, counting, place value); Key Math, Revised: Geometry (shapes, patterns, specialized terms); Key Math, Revised: Problem Solving (word problems); Woodcock-Johnson, Revised: Calculation (Woodcock and Johnson, 1989: mixed written calculation problems); Number Knowledge (Case et al., 1996); Word Problems (Okamoto, 1992); Counting Span (Case, 1985); **Verbal Measures**-Stanford-Binet IV: Vocabulary (definitions); Stanford-Binet IV: Comprehension (practical reasoning); Stanford-Binet IV: Memory for Sentences (immediate repetition of sentences); **Visuo-spatial Measures**-Stanford-Binet IV: Pattern Analysis (copying designs with patterned cubes); Stanford-Binet IV: Matrices (choosing which of 5 alternatives would complete 2 × 2 and 3 × 3 matrices; deciphering letter placement in complex matrices); Visuo-spatial Span (Crammond, 1992, measure visuo-spatial WM).
22	O'Boyle et al., 2005	**Visuo-spatial Processing Measure:** 3-dimensional object mental manipulation task.
23	Lubinski and Benbow, 2006	**Math Ability Measure:** SAT-M; **Verbal Ability Measure:** SAT-V; **Spatial Ability Measures:** mechanical reasoning & space relations assessments; **Values Assessments:** the theoretical, aesthetic, social, economic, and religious subtests of the Study of Values (Allport et al., 1960); **Preferences Assessments:** the realistic, investigative, artistic, social, enterprising, and conventional subtests of the Strong-Campbell Interest Inventory's RIASEC (Harmon et al., 1994).
24	Wai et al., 2009	**Math Ability Measures:** mathematics information test, arithmetic reasoning, introductory mathematics, advanced mathematics; **Verbal Ability Measures:** vocabulary, English composite, reading comprehension; **Spatial Ability Measures:** three-dimensional spatial visualization, two-dimensional spatial visualization, mechanical reasoning, abstract reasoning.
25	Desco et al., 2011	**Math Reasoning Measures:** mathematical reasoning task; **Other Non-verbal Reasoning Measures:** Tower of London test and Raven's Advanced Progressive Matrices.
26	Hu et al., 2011	**WM Measure:** Forward digit span and letter span; **General IQ Measure:** WISC-R.
27	Wei et al., 2012	**Basic Numerical Processing Measures:** Comparison of dots of two arrays [adapted from Test of Early Mathematical Ability-2 (TEMA-2), (Ginsburg and Baroody, 1990)]; comparison of colored dots (adapted from Halberda et al., 2008); estimation of numerosity (adapted from Butterworth, 1999); number comparison task (Number Stroop Task, Zhou et al., 2007); **Complex Numerical Processing Measures:** multiple-digit computation (author designed); number series completion (Cognitive Abilities Test 3, Smith et al., 2001); arithmetic learning (adapted from Delazer et al., 2005); **Spatial Processing Measures:** Three-dimensional mental rotation (adapted from Shepard and Metzler, 1971); spatial WM (similar to Corsi Block task, Corsi, 1973); figure analysis test (adapted from Cognitive Ability Test level G); **Language Measures:** Word rhyming (adapted from Tan et al., 2001, 2003); word semantic processing (adapted from Siegel and Ryan, 1989; So and Siegel, 1997); sentence syntactic processing (adapted from Hagoort et al., 1993); word paired-associate learning (adapted from Calkins and Skelton, 1894); **General Cognitive Measures:** Simple reaction time task (adapted from Butterworth, 2003); attention (adapted from Fan et al., 2002); abstract reasoning (Raven's Progressive Matrices).
27	Hoppe et al., 2012	**Visuo-spatial Processing Measure:** 3-dimensional object mental manipulation task.
28	Van Garderen, 2016	**General IQ to Sort Participants:** WISC-R; **Math Ability Measures:** Calculation, Math Fluency, and Applied Problems subtests of the Woodcock-Johnson, Mathematical Processing Instrument (Hegarty and Kozhevnikov, 1999), math word problems; **Visuo-spatial Ability Measures:** MGMP-SVT (citation of this measure not given in study), WISC-III Block Design subtest.
29	Sella et al., 2016	**Math Ability Measures:** number line paradigm (primary task), numerical Stroop, numerical agility; **Verbal Reasoning Measures:** Vocabulary subtest of the WASI-II (Weschler Abbreviated Scale of Intelligence; Wechsler and Chou, 2011); **Non-verbal Reasoning Measures:** Similarities, Block Design, and Matrix Reasoning subtests of the WASI-II.
30	Amalric and Dehaene, 2016	**Math and Verbal Processing Measure:** fast semantic judgment task for mathematical and non-mathematical statements.
31	O'Boyle et al., 1991	**Emotional Processing Measure:** Chimeric face judgement task; **Verbal Abilities Measure:** noun/verb judgment task.
32	O'Boyle et al., 1994	**Motor, Verbal, and Spatial Ability Measure:** finger-tapping task with verbal and spatial interference load conditions.
33	Krause and Heinrich, 2003	**Math Ability Measure:** geometry problem (by calculation or imagery).
34	Minati and Sigala, 2013	**Calendrical Ability Measure:** calendar dates task.
35	Zhang et al., 2014	**Reasoning Ability Measure:** syllogism validation task.

### Environment, motivation, and practice

Some studies have shown positive correlations between mathematical giftedness and individual motivation. Kennedy and Walsh (1965) conducted a study with gifted high school students in which they used a Principal Component Analysis (PCA) of 33 variables to compare 15 cognitive/personality factors (eight assessments were used; see Table 1; Row 2). The mathematically gifted students (*n* = 90) were selected based on their ability and also for showing high interest in math, while the other groups (*n* = 63) showed high general cognitive ability but were not specifically gifted nor interested in mathematics. The authors concluded that mathematical giftedness correlated with high general intellectual ability combined with a high drive to succeed, authoritarian attitudes, and a lack of involvement in social, interpersonal, or religious issues. One potential confound to note in this study is that the correlation with high motivation may be the result of the authors recruiting participants based on not only high math ability but also high interest in mathematics.

Some studies with math prodigies also point to the importance of practice and motivation. Jensen (1990) having attended an impressive demonstration by Shakuntala Devi (in 1988 when she was 59 years old), invited her to his laboratory in Berkeley, to measure various aspects of her cognitive abilities. Since the time she was 3 years old, Devi had been performing remarkable feats of mental calculation on stage, which included such things as multiplying and finding roots of very large numbers. As one example, Jenson reports seeing Devi mentally find the 23rd root of a 201-digit number in 50 s. A battery of assessments were given to Devi which consisted of the Raven Advanced Progressive Matrices (Raven et al., 1998), the forward and backward digit span subtests of the Wechsler Intelligence Scale for Adults (WAIS; Wechsler, 1997), as well as five different elementary cognitive tasks (ECT) to measure her speed of information processing. The first ECT was a simple reaction time task in which she pressed a button as quickly as she could when a light appeared. The second ECT was a choice reaction time task. In this, the light on the screen appeared in eight different locations, which corresponded to eight different buttons. Devi was instructed to press as soon as she perceived the light. Utilizing the same format as above, the third ECT was an odd-one-out paradigm in which three lights would appear simultaneously with two of them being closer to each other than the third, which was the “odd” one. The task was to press the button corresponding to the location of the “odd” light as quickly as possible. The fourth and fifth ECTs were a visual search task and a memory search task, respectively. In the former, a single digit would appear on a computer monitor for 2 s. After an interval of 1–4 s, a series of digits (set size 1–7) would appear. Devi was to press “Yes” or “No” as quickly as possible depending on whether she observed the initial presented digit in the second presented set. The memory search task was similar but reversed in that the set of digits was presented as the first stimulus. Analyses of the above tasks, compared with numerous others who had taken these same tests in Jenson's lab, revealed that Devi did not show any exceptional abilities in her general cognitive skills, including her working memory (WM). Jenson concluded that her abilities may, in part, be due to better-than-normal ability to encode into long-term memory. This along with her love of numbers and the inordinate amount of time she spent as a young child playing with them, and perhaps committing large volumes of numerical information to memory are, he reasons, the best explanation for her capabilities.

Wu (1996) collected data from adolescents in the Math Olympians program (*n* = 36), utilizing questionnaires and in-depth interviews, in order to determine which family, school, and Math Olympiad program factors contributed to their mathematics development. It was concluded that high socio-economic status (SES) (determined by the father's higher than average occupational status, income, and education level), and family involvement (more positive and supporting then average) were the factors that most correlated with students' math ability. High math ability was assumed by the math Olympiad status. No cognitive variables were measured in this study.

Fehr et al. (2010) conducted a study with a prodigious mathematician and a group of normal controls (*n* = 11). Participants engaged in four basic arithmetic operations with 1 or 2-digit numbers during fMRI measurement. They categorized the arithmetic problems as moderate or difficult, with the latter being those with either borrowing or carrying required. Results showed that the brain regions activated by the mathematical prodigy were similar to controls in both the moderate and difficult math problems. From this they concluded that his prodigiousness was most likely due to diligent practice in math over the course of his life rather than to special general cognitive abilities.

Another fMRI study by Fehr et al. (2011) explored the neural correlates of calendrical calculation experts with two adults—one a prodigy with Asperger's Syndrome and the other a self-taught mathematical prodigy. Fifty-six future and past dates (1100–1800 and 2200–2800) were presented for 2,000 ms (e.g., “July 25, 1289”) after which the subjects were shown four possible weekdays. They were to select via key press the day, of those presented, which could match the calendar date. If mathematical giftedness were correlated with a modular neural network, the authors expected that these two subjects, representing two different types of high mathematical performance, would display similar brain activity. In contrast to this, there were considerable individual differences in activation patterns. The authors concluded that giftedness in complex mental processing may be driven by a history of intensive practice as well as by the strategy chosen.

### Logical reasoning and fluid intelligence

Some studies have identified factors related to logical reasoning or fluid intelligence (the ability to solve novel problems, identify patterns and use logic in novel situations) as fundamental to mathematical giftedness. One such study was conducted by Prescott et al. (2010). Using groups of mathematically gifted (*n* = 8) and control (*n* = 8) adolescents, they explored brain connectivity by presenting the subjects with 3-dimensional objects composed of 10 blocks. One object was shown at the top of the screen along with four objects along the bottom of the screen. The subjects were to mentally rotate each of the four objects in order to determine which could match the object shown at the top. Structural equation modeling analysis of fMRI data showed heightened intrahemispheric fronto-parietal connectivity and enhanced interhemispheric frontal connectivity between dorsolateral prefrontal cortex and premotor cortex. The authors suggest that these are the main neural characteristics of the math gifted brain, in which intrahemispheric fronto-parietal connectivity is more specific to mathematical aptitude/talent, and the anterior subnetwork that includes dorsolateral prefrontal cortex, premotor regions, and the anterior cingulate can be connected to general intelligence.

In a study with children who showed high (*n* = 14), average (*n* = 16), and low (*n* = 13) mathematical performance, Morsanyi et al. (2013) administered eight tests to measure IQ, WM, reading skills, and reasoning ability (Table 1; Row 8). To measure reasoning they used transitive inference problems in which there were four problems with believable conclusions (e.g., “Insects are smaller than rabbits”), four with unbelievable conclusions (e.g., “Rabbits are smaller than insects”), and four with conclusions that were belief-neutral (e.g., “John is bigger than Tom”). The children were instructed to pretend that the premises were true, even if they sounded “funny or strange.” The authors reported that the ability to reason independently of one's beliefs corresponded most significantly with both high and low mathematical abilities.

In an EEG study, Zhang et al. (2015b) investigated reasoning ability with respect to the relationship between executive function and the dynamic frontoparietal network. They tested math gifted adolescents (*n* = 11) and normal controls (*n* = 13) in verbal reasoning tasks in which subjects were presented with a major premise, a minor premise, and then a conclusion. The samples could be valid (e.g., No A is B, All X are B, No A is X) or invalid (e.g., Some D are E, All K are D, Some K are E). The subjects were to verify the validity of the arguments by key press. The authors conducted cortical source analysis of the EEG theta band data which showed stronger responses, signifying stronger neural effort, with math-gifted participants. The authors concluded that the brains of math gifted subjects invested a greater amount of cognitive resources in order to temporarily create an enhanced mental “workspace” which facilitated easier solutions to deductive reasoning problems.

### Short-term memory

Dark and Benbow (1990) compared a group of mathematically talented adolescents (*n* = 20) with a group of normal controls. If a significant effect were found on a certain measure, the mathematically talented group would also be compared with a group of verbally talented adolescents (*n* = 20) and a group of college-age students (*n* = 20). In Experiment 1, the two measures used were the following: (1) equation stimuli—the participants were to write number equations to represent what was expressed in verbal sentences, and (2) algebra story problems. In Experiment 2, the same groups of subjects were measured with the following tasks: digit span, spatial span, letter-digit continuous paired associates, and letter-location continuous paired associates. Experiment 1 showed that the mathematically talented group outperformed all three of the other groups in translating verbal expressions into numeric equations. For the algebra problems they performed better only than the normal control group. Experiment 2 showed that the mathematically talented group outperformed the normal control and verbally talented groups, and equal to the college-age group, in regards to short-term memory involving digits.

### Working memory

Several studies have also found correlations between WM and mathematical giftedness. Dark and Benbow (1991) examined three aspects of WM—encoding, capacity, and manipulation of information—by varying two types of stimuli (numeric and verbal) with two groups of highly gifted 13–14 year old participants [mathematically (*n* = 22) and verbally (*n* = 19) precocious]. The first type of assessment given consisted of four span tasks (digits, letters, words, and locations) in which five lists were presented each trial at each of four lengths. The participants were to recall each of the lists, which were only counted correct if recalled completely accurately. The second type of task administered was a continuous paired-association task, which utilized digits, letters, and locations. For this, participants were shown sets of items of which one was associated with an item of another type (for example one of the letters may have been associated with a digit such as F = 9). They were to encode and correctly recall the association for each trial. Post-analysis, the authors found that visual WM encoding speed was more related to mathematical precocity than verbal precocity. In regards to capacity and manipulation of information, the math precocious group only scored higher with digits and location stimuli while the verbally precocious group showed enhanced WM capacity when words were used as stimuli.

Dark and Benbow (1994) conducted another study with adolescents (*n* = 21) who were either mathematically or verbally precocious (the study does not make clear how many were in each group). The participants engaged in the following tasks: simple categorization (classify a presented stimulus as quickly as possible), dual-task recall (verbal categorization made while maintaining a five-item list in memory), and continuous paired-association (remember which letters are paired with which number). The authors concluded that high performance in verbal working memory is more related to mathematical precocity than verbal precocity.

A PET study contrasted a calculating prodigy with a group of non-experts in calculation (*n* = 6) (Pesenti et al., 2001). The task consisted of solving a pair of arithmetic problems each trial—one to be solved by calculation and the other by memory retrieval. The calculating prodigy was presented with a 2-digit squaring problem for the memory retrieval (e.g., 73 × 73) and 2-digit multiplication problems for calculations (e.g., 68 × 76). The control group was presented with simple multiplication facts for memory retrieval (e.g., 5 × 9) and 2-digit multiplication problems with products less than 1,000 for calculation (e.g., 25 × 13). The fMRI analysis revealed that a number of brain areas were activated during calculation but not during memory retrieval. Of these, only the following were observed in the prodigy but not in the controls: left paracentral lobule, right middle occipito-temporal junction, right medial frontal gyrus, right anterior cingulate gyrus, and the right parahippocampal gyrus. The authors concluded that the calculating prodigy used different brain areas for calculation than did the controls. They further concluded that the prodigy's calculating skill was due to being more efficient at switching from effortful WM storage strategies to efficient episodic storage and retrieval. Here it is important to note that the conclusions drawn (and similar conclusions from other studies) constitute invalid “backwards inference” from brain imaging data (Poldrack, 2006). Backwards inference in neuro-imaging refers to the attribution of a specific cognitive process based on neuro-imaging results, where this cognitive process has not itself been measured within the study. It is a fallacy because neural regions do not tend to be domain-specific; that is, one region may be activated during many cognitive processes, thus activation of the region cannot be taken as evidence for any of these processes individually. This will be considered in the Discussion.

Tanaka et al. (2002) tested abacus experts (*n* = 10) and controls (*n* = 13). They used event-related fMRI and a delayed match-to-sample task using digits as stimuli. Participants were shown one set of numbers followed by another. Their task was to determine whether the second set was the same or different from the first set. Results showed greater activation for the expert group in the bilateral superior frontal sulcus and the superior parietal lobule, which are believed to be correlated with visuo-spatial WM. The authors concluded that their study showed that abacus experts, more so than controls, utilize a visuo-spatial representation for digit memory. However, this conclusion not only represents backwards inference but also, similarly to other studies above, has not tested for stimulus and task specificity of brain imaging effects. Hence, first it is not clear whether the observed activation differences are related to the abacus task at all. Second, observing certain brain activation in this task cannot be interpreted in functional (brain activity) terms if the specific function of these areas has not been determined in the study (Poldrack, 2006).

Swanson (2006) administered a battery of tests (see Table 1; row 15) to 6–8 year old mathematically precocious children (*n* = 50) and normal controls (*n* = 77). The assessments measured three components of WM (phonological loop, visuo-spatial sketchpad, and central executive) as well as naming speed, random generation, and letter fluency. In addition to standardized tests, participants were also administered several experimental tasks. In a word recognition task, children were presented with lists of words of increasing difficulty. They were asked to read the words until 10 consecutive errors occurred. There were also two naming speed tasks. For digit naming speed, the children were shown two arrays with 36 digits each which they were to name as quickly as possible while being timed. For letter naming speed the procedure was the same but with letters instead of digits. To measure verbal recall with central executive WM, a Listening/Sentence Span task and a Digit/Sentence Span task were utilized. In the former, children attempted to read and understand a passage while remembering the last word in each sentence. In the latter, children would attempt to remember numerical information embedded in a sentence while also trying to understand the meaning of the sentence. To measure the recall of visuo-spatial information with central executive WM, a visual matrix task and a mapping task was used. In the former, children were briefly presented with a series of dots in a matrix. After they disappeared, the participants were asked if dots were present in a particular column to which they would respond “yes” or “no.” In the later task, children were given one of four different strategies for finding directions on a map. After this, they were briefly presented with a map after which they were asked to process questions about details on the map (such as whether there was a traffic light on a particular street). The results showed that the mathematically precocious children performed better than the mathematically average children on the inhibition, naming speed, and the central executive WM tasks. No significant difference was found between the groups in regards to the visuo-spatial tasks.

In a study with 10–12th grade students, Leikin et al. (2013) used the Ravens Advanced Progressive Matrices Test (Raven et al., 1998) and the Mathematics subtest of the Scholastic Achievement Test (SAT) to divide the participants into four groups—generally gifted and excelling in mathematics (*n* = 34), generally gifted and not excelling in mathematics (*n* = 41), not generally gifted and excelling in mathematics (*n* = 36), and not generally gifted and not excelling in mathematics (*n* = 46). They administered the Digit Span and Working Memory for Digits and Letters subtests of the Wechsler Intelligence Scale for Children (WISC-III; Wechsler, 1997) as well as a visuo-spatial WM task in which the researcher would point at 10 wooden blocks one at a time at a rate of 1 block per second after which participants attempted to recall the correct sequence the blocks were pointed at. The authors concluded that general giftedness is related to high verbal short-term memory (for both the phonological loop and phonological central executive mechanisms) while excellent ability in mathematics was concluded to relate to high visuo-spatial WM.

Similarly, Leikin et al. (2014) conducted a study with 10–11th grade students who were divided into three groups—super math gifted (those who hold the top level of giftedness (Silverman, 1989) (*n* = 7), generally gifted and excellent in math (*n* = 26), and not generally gifted and excellent in math (23). Using the same measurements as described in their 2013 study above, the authors found that the super math gifted students displayed superior performance on the visuo-spatial WM tasks as well as the pattern recognition—an indicator of visual perception.

Ruthsatz et al. (2014) compared the cognitive profiles of child prodigies (*n* = 9) across the domains of art, music, and mathematics. The children were considered to be prodigies if they had achieved acclaim in their area by adolescence. The participants were tested using the Stanford-Binet, 5th ed. test battery. The results showed distinct cognitive profiles among the child prodigies for each domain. The music and math prodigies scored significantly higher in WM skills (both verbal and non-verbal WM) compared to the art prodigies. The math prodigies scored the highest in general intelligence and visuo-spatial skills, while art prodigies scored much lower than the others in visuo-spatial skills. The authors reported all groups as showing exceptional long-term memory.

In a combined neuroimaging and behavioral Zhang et al. (2015a) tested adolescents—math experts (*n* = 8) and normal controls (*n* = 7)—who were given tasks with varying cognitive demands. The first task involved inducing and applying an arithmetic rule (addition or subtraction) when presented with three triangles, each of which contained three single-digit numbers inside at each vertex. Two of the triangles were presented first to the left of an arrow. By observing the three numbers inside each of the triangles, the subjects were to use induction to formulate the arithmetic rule which was being used, for example, A+B = C or A+C = B. The subjects would be shown two of such triangles on a computer screen to the left of an arrow. Upon being presented with the third triangle, the subjects were asked to verify whether the rules were consistent. The second, more difficult, task was similar to that described above while using squares with four numbers inside instead of triangles with three. Using four numbers greatly increased the possible rules that could be induced. Three “mental state” factors were analyzed—mathematical ability, task complexity, and short-term learning. Overall, the math gifted subjects showed a more rapid decrease of brain activation over the task course than the control group, due to a stronger short-term learning effect. The authors suggest that this might be related with reducing cognitive load from task demands in working memory while a more efficient problem-solving strategy is developed.

In a study with elementary school students, Barner et al. (2016) trained children to use a mental abacus to determine if such training, which they believed primarily involved training spatial WM, could improve mathematical abilities. Over a period of 3 years all of the children in the study participated in their school's normal mathematics curriculum. In addition to this, the experimental group (*n* = 100) received 3 h per week of extra training (both physical and mental) with an abacus, while the control group (*n* = 104) received the same amount of extra practice with the *Enjoying Mathematics* curriculum from Oxford Press. In order to assess the children's performance, a battery of tests was given to the children at the commencement of the study for baseline data and then annually at the end of each school year. For mathematics measures, the Woodcock-Johnson Tests of Achievement and the Math Fluency subtest of the WIAT were used. For cognitive measures, they administered the Raven's Progressive Matrices (Raven et al., 1998) and a number comparison task in which the children chose the larger of two dot arrays. They also administered the following non-standardized tasks which were not described in the paper: a mental rotation task, an adaptive test of short-term memory, and an adaptive test of visual WM. Results showed that the mental abacus training group significantly improved in mathematics ability compared to the control group; however, no improvement in domain-general cognitive functions was observed. Rather, higher spatial WM scores during the baseline pre-tests correlated with improved learning during the mental abacus training. The authors concluded that higher spatial WM “mediated” mental abacus learning (p. 8).

### Visuo-spatial abilities

Several studies have found a connection between visuo-spatial abilities and mathematical giftedness. In one, Benbow and Minor (1990) recruited 13-year-olds (*n* = 144) who were precocious in either mathematical reasoning ability (*n* = 106), verbal reasoning ability (*n* = 20), or both (*n* = 18). Analysis of the results of a battery of assessments (Table 1; Row 20) showed that students who were gifted in verbal reasoning performed better in verbal and general knowledge tests, while students gifted in mathematical reasoning scored higher in non-verbal reasoning, associative memory, and spatial ability.

In a study by Robinson et al. (1996), 778 preschoolers and kindergartners were recruited based on being identified by their parents as advanced in mathematical reasoning. The children were given two arithmetic subtests of the Cognitive Abilities Test. Then the children who scored high on these (*n* = 310) were given several additional measures (Table 1; Row 21). A confirmatory factor analysis revealed that the scores on the visuo-spatial subtests were highly correlated with those measuring mathematical achievement (girls: coeff = 0.73, Z = 8.60; boys: coeff = 0.76, Z = 10.07).

O'Boyle et al. (2005) also administered a mental-rotation task to mathematically gifted adolescents (*n* = 6) and controls (*n* = 6) in an fMRI study. In this task, the participants were simultaneously presented with a target 3-dimensional object and four probe 3-dimensional objects. The objects were composed of 10 blocks and were randomly assigned different arrangements. The participants were to mentally rotate the target in their minds in order to determine which of the four probes it could be. The authors suggested that the mathematically gifted adolescents displayed activation of a qualitatively different neural network than control participants. A strong caveat to consider is that there were very few participants in this study. Also important to consider is that the authors did not test for stimulus specificity (as in the study above), nor for task specificity of brain imaging effects—i.e., observing certain brain activation in this task cannot be interpreted in functional (brain activity) terms if the function of these areas has not been determined within the study (Poldrack, 2006).

Lubinski and Benbow (2006) analyzed results from the Study of Mathematically Precocious Youth (SMPY), which was founded by Stanley (1996) at Johns Hopkins University and is currently co-directed by Benbow and Lubinski at the Peabody College of Vanderbilt University. SMPY is a 50-year longitudinal study utilizing five cohorts and consisting of over 5,000 intellectually talented individuals. In this study, subjects were identified and assessed at age 12–13, in four cohorts, using the top 1–3% (depending on the cohort) of the math and verbal subtests of the SAT. Subjects were also assessed on spatial ability using two differential aptitude tests, mechanical reasoning and space relations, as well as on values and academic preferences (Table 1; Row 23). The subjects were then assessed for various outcomes over several decades including the following: favorite & least favorite high school course, college major, occupation, income, and whether (and in what subject) a Ph.D was earned. An analysis of the cognitive and emotional factors across each of the outcomes revealed that spatial ability was the most predictive factor of involvement in and success in mathematics and science careers, adding a 2.4% incremental predictive validity.

Following on the above study, Wai et al. (2009) investigated the importance of spatial ability in regards to educational and occupational pursuits, with particular attention on STEM (science, technology, engineering, and mathematics) domains. They recruited subjects from the Project Talent pool and compared these results with the NSF report and SMPY project databases, which in total ranged in years from the early 1950s to 2009 and included over 400,000 high school students. Subjects were assessed on mathematical, verbal, and spatial ability (Table 1; Row 24). From the data, the authors argued that spatial ability plays a critical role in the development of expertise in mathematics and other STEM disciplines.

In an fMRI study (Desco et al., 2011), mathematical reasoning tests were administered to mathematically precocious (*n* = 13) and typical adolescents (*n* = 14). The Tower of London test was administered to measure executive function, while Raven's Advanced Progressive Matrices (Raven et al., 1998) was utilized to measure fluid reasoning. The results showed more activation for mathematically precocious students in the right inferior parietal lobule, the anterior cingulate gyrus, and frontal areas—brain areas often thought to be associated with visuo-spatial processing. As in several other studies, it is important to note that the conclusions drawn here constitute invalid “backwards inference” from brain imaging data which will be considered further in the discussion.

Hu et al. (2011) conducted a diffusion tensor imaging (DTI) study with children who were abacus experts (at least 3 years of practice with abacus; *n* = 25) and controls (no abacus experience; *n* = 25). They administered a Forward Digit/Letter Span task to the children in which several sequences of single digits or letters were aurally presented at a rate of about one per second. The children were to recall and orally name the digits or letters in the order in which they were presented. The reading subtest of the WISC was also administered to assess intelligence. Behavioral results showed that the children who had long-term abacus mental calculation training had larger digit and letter memory spans (thought to be measures of short-term memory) than controls. In regards to DTI, the children with abacus training showed an increase in white matter connections between areas thought to support motor functions and visuo-spatial processing—the corpus callosum, the left occipitotemporal junction, and the right premotor projection. Note that this is another example of using “backwards inference” in regards to the conclusions from the neural imaging data.

Research using adult participants has also explored spatial abilities and mathematical giftedness. Wei et al. (2012) with Chinese college-age adults (*n* = 80) investigated associations between performance on an advanced mathematics assessment and a battery of 17 cognitive tasks (Table 1; Row 27). In the main task, 18 advanced mathematical concepts were introduced to participants (all of which had not yet been learned by these participants but which came from a textbook being used by mathematics majors at the university). Participants read a passage introducing each concept and answered two multiple choice questions per concept. Analysis revealed that proficiency in advanced mathematics was correlated with high spatial abilities but not with basic numerical processing or computation.

In a combined behavioral and fMRI study (Hoppe et al., 2012) math gifted adolescents (*n* = 17) and controls (*n* = 20) completed a mental rotation task. They were first briefly presented with an L-shaped three-dimensional object composed of three blocks on the computer screen. Next, four different arrows would appear in sequence which signaled to the participant in what direction they were to mentally rotate the object. Finally, another three-dimensional L-shaped object was shown and the participants verified by key press whether or not it matched what the object should look like after the mental rotations they were instructed to carry out. From this the authors concluded that the math gifted students showed superior spatial ability relative to controls as well as that activation of the inferior parietal lobule correlated with mental rotation performance. However, one thing to note is that the authors did not employ a spatial assessment to measure for stimulus specificity. In other words, they assumed the mental rotation task measured spatial ability without determining this within the study.

Van Garderen (2016) conducted a study with 6th grade students with the aim to explore the relationship between visual imagery and visuo-spatial ability while students were engaged in mathematics word problems. She utilized the WISC-R to divide the children into three groups—math-gifted (*n* = 22), math-typical (*n* = 22), and below average math (*n* = 22). This study was conducted in two sessions. During the first, the Calculation, Math Fluency, and Applied Problems subtests of the Woodcock-Johnson were administered, as well as the Middle Grades Mathematics Project Spatial Visualization Test (MGMP-SVT; Lappan 1981) to measure visuo-spatial ability. During the second session, participants completed the WISC-III Block Design subtest, also to measure visuo-spatial ability, as well as the Mathematical Processing Instrument (Hegarty and Kozhevnikov, 1999) to measure visual imagery while performing math word problems. The author reported that the mathematically gifted students showed better visuo-spatial ability than students with typical or low mathematical ability.

An adult study by Sella et al. (2016) aimed to investigate whether basic numerical skills are correlated with more complex arithmetical abilities. They recruited researchers to participate (Ph.D. students or post-doctoral) who were either mathematicians (*n* = 19) or a highly intelligent group of non-mathematicians (Humanities; *n* = 19). The primary task was a number line paradigm in which participants were presented on a computer screen with a horizontal blue line. The line was to represent a number line with the range, from left to right, of −100 to 100. Fifteen positive and 15 negative numbers were shown on the screen. After this, each of the participants were to click on the line to represent where the number should be located. The second task, numerical Stroop, involved being presented with pairs of digits which could differ both in numerical value and physical size. There were two conditions, congruent (the numerical size matched the physical size) or incongruent (the numerical size did not match the physical size). The subjects were to choose the larger number based on physical size, ignoring numerical value. A third task, numerical agility, was administered in which the subjects were to repeatedly generate the number 24 from four presented numbers, using only the four basic arithmetic operations. Finally, the subjects were also given the Vocabulary, Similarities, Block Design, and Matrix Reasoning subtests of the Weschler Abbreviated Scale of Intelligence (WASI-II; Wechsler and Chou, 2011) to measure verbal and non-verbal IQ. In regards to the primary number line task, results showed that the mathematicians performed significantly better at mapping positive numbers on the mental number line and that it could be predicted who was in the mathematics group based on their performance. Initially, in the ANOVA analysis it appeared that these basic number mapping skills were mediated by more advanced mathematical skills; however, when a mediation model analysis was conducted which included the results of the Block Design subtest, to measure visuo-spatial skills, the mathematician's superior performance on the number line task was concluded to be better explained by non-numerical visuo-spatial skills.

Amalric and Dehaene (2016) conducted an fMRI study with professional mathematicians (*n* = 15) and non-mathematicians of matched academic standing (*n* = 15). The participants performed fast semantic judgments on both mathematical and non-mathematical statements. The authors found that there was no significant brain activation in areas believed to be associated with verbal processing in the mathematical statement condition with the professional mathematician group. Rather, the activated areas were believed to be associated with space and number—bilateral frontal, intraparietal, and ventrolateral temporal regions. However, it should be noted that, as in a few other studies, this conclusion is based upon “backwards inference” as the function of the detected areas was not tested in the study. In addition, no functional connection has been shown between the putative cognitive functions carried out in the detected brain areas and mathematical ability. Therefore, conclusions are based on invalid inference and many untested assumptions.

### Other neuro-imaging studies

Several neuroimaging studies explored the neural correlates of mathematical giftedness. For example, an EEG study was conducted by O'Boyle et al. (1991) in order to determine if hemispheric alpha-band activity differs between mathematically precocious youth (*n* = 6) and controls (*n* = 8). The task consisted of (1) a period of rest with the eyes closed (for measuring baseline brain activity), (2) judging which of two chimeric faces (top or bottom) appear happier, and (3) determining whether a presented word is a noun or a verb. In regards to the alpha-band analysis, at baseline the left hemisphere of the math-prodigious group was more active across all lobes (frontal, temporal, parietal, and occipital). For the chimeric face processing, the math-prodigious group showed significantly reduced activity at the right temporal lobe. Finally, for the noun/verb determinations there were no significant alpha reductions for either group. The authors believed that these electrophysiological findings confirmed previous behavioral results showing that the involvement of the right hemisphere during cognitive processing tasks may correlate with mathematical precocity.

A study by O'Boyle et al. (1994) explored the brain structure of math gifted adolescents (*n* = 24) and controls (*n* = 16) using a finger-tapping paradigm. The participants were to tap a key as quickly as possible while either sitting silently (no load), encoding by reading a paragraph aloud (verbal load), or encoding a randomly presented form into memory (spatial load). The spatial load condition showed a slight reduction in tapping rate for both hands in both groups; however, in the verbal load condition, the tapping rate of the controls decreased only with the right hand while it decreased in both hands for the math gifted group. That the math gifted subjects showed increased reliance on the right hemisphere for verbal tasks, contrasted with the controls, confirmed previous studies concerning right hemisphere functioning during cognitive tasks being a correlate of mathematical giftedness.

Krause and Heinrich, (2003) used event-related synchronization (ERS) of EEG data with instantaneous coherence analysis to measure entropy reduction. That is, they used ERS to detect synchronization states (called “microstates”). They then measured the strength of the synchronization between these microstates, which was believed to show the level of entropy (i.e., disorder of thought). The authors contend that there is an inverse relationship between entropy and the strength of synchronization connecting microstates. In this study, math gifted (*n* = 12) and control (*n* = 12) adolescents were tasked with solving a geometry problem using either calculation or imagery. The authors reported that the math gifted group showed higher entropy reduction than controls. The results of differences in performance between task conditions were not reported. This study built upon a previous experiment using only a math gifted group (Krause et al., 2001), in which the authors reported that entropy reduction could be measured with math gifted individuals.

Minati and Sigala (2013) used fMRI to investigate the neural basis of expert calendrical skills with a gifted adult calculator. In the task, the subject was given dates from three periods of varying remoteness. The dates were presented in the form of a true or false question such as “07-Feb-1972 is a Monday; L: true, R: false?” which the subject would answer with a key press. Analysis of the fMRI data revealed higher activation in occipital and medial-temporal areas for the processing of well-practiced close dates while showing more frontal, orbitofrontal, and anterior cingulate connectivity for less-practiced remote dates. The authors concluded that complex calendrical calculation ability may be initially supported by extensive attentional and strategic resources, and then followed by being gradually replaced by access to long-term memory for more practiced material as expertise develops. This study, like others discussed, bases its conclusions on reverse inference of brain imaging data.

Another EEG study by Zhang et al. (2014) utilized dynamic network analysis of gamma band event-related synchronization (ERS) to compare performance on a deductive reasoning task between math gifted adolescents (*n* = 11) and age-matched controls (*n* = 13). The participants were presented with syllogisms, consisting of a major premise, a minor premise, and a conclusion, which were either valid or invalid. They were tasked with judging the validity of the syllogisms via key press. For the behavioral results, the authors reported that the math gifted group was faster to report the answers, but there was no significant difference in accuracy between the groups. Regarding the dynamic network analysis, the authors reported that the math gifted group showed a more highly integrated fronto-parietal network, based on prolonged ERS activity in these regions as compared with the control group.

Three neuroimaging studies conducted with adolescents did not have a functional task but rather were concerned only with exploring differences between groups of participants whose brains were in a resting state. In the first, Alexander et al. (1996), in an EEG study, investigated the eyes-open resting state for the alpha band between math gifted adolescents (*n* = 30), control adolescents (*n* = 30), and college students (*n* = 30). The authors reported that the overall alpha resting potential was significantly greater in the adolescent control group as contrasted with the other two. There were no differences between math gifted adolescents and college-age students; however, there were differences in left/right hemisphere activation patterns. The authors suggest that these results may show an enhanced brain developmental state for the math gifted adolescents. It is important to note here that lower overall alpha resting potential could have been driven by different factors in math gifted adolescents as compared with the college students (e.g., high drive for math gifted and greater sustained attention for older college students). This would fit well with the different hemispheric activation patterns between the groups.

The second “resting state” study, a diffusion-tensor imaging study by Navas-Sánchez et al. (2013) explored the white matter structure differences between math gifted adolescents (*n* = 13) and age-matched normal controls (*n* = 23). An assessment of IQ was given using the Vocabulary, Information, and Block Design subtests of the WISC-Revised. A T1-weighted MRI scan and diffusion-weighted scans were taken of the participants at rest. The analysis of the IQ assessments showed that the math gifted group had a significantly higher score across all three measures. The analysis of the white matter microstructures showed increased fractional anisotropy (FA) for the math gifted group in the corpus callosum which was dependent on IQ scores. Increased FA independent of IQ was also observed in frontal-parietal and frontal-striatal association tracts for the math gifted group. The authors concluded white matter organization may be different for the math gifted and that information transfer across hemispheres may be crucial to higher intelligence in general.

In the third “resting state” study, Navas-Sánchez et al. (2016) conducted an MRI study with math gifted (*n* = 13) and normal control (*n* = 17) adolescents. While the subjects lay passively, a T1-weighted scan was performed using a 1.5 Tesla MRI. The data were pre-processed by skull stripping and tissue segmenting, and using a vertex-wise analysis to contrast group differences in whole-brain cortical thickness and surface area. Results showed that the math experts presented a thinner cortex and a larger surface area in the frontal-parietal area as well as in areas key to executive processing and creativity. Specifically, in the math experts group lower cortical thickness was measured in the following areas: Superior parietal right hemisphere (RH), Superior frontal RH and left hemisphere (LH), Pars orbitalis (LH), and precuneus (LH); while greater surface area was measured in the following areas: Superior frontal (RH and LH), Lingual (RH), and Inferior parietal (LH).

## Discussion

We have reviewed currently available psychological and cognitive neuroscience studies on mathematical giftedness. At the moment relatively few studies have focused on this topic and the variables measured within each study are widely diverse. This does not allow us to carry out a principled effect size analysis. However, existing studies point to variables worthy of further exploration and some problematic aspects of the literature in need of correction. We deliberately keep theoretical speculations at a minimum as a great number of alternative models can be imagined on the basis of the current weak evidence base.

### Heterogeneous populations

Studies tested various categories of math gifted individuals. For example, some participants in the studies above are “lightning” calculators, while others are skilled at sophisticated mathematical reasoning. In some instances, individual math prodigies participated in a study in the absence of any control group (Fehr et al., 2011; Minati and Sigala, 2013). In many studies, math gifted groups were selected based on performance in school or on mathematics tests, which usually require a combination of both high calculation and mathematical reasoning. This heterogeneity probably contributes to the variability of findings discussed below.

### Heterogeneous findings with focus on visual (memory) processes

In line with the heterogeneous nature of mathematical disabilities (e.g., Rubinsten and Henik, 2009; Fias et al., 2013), mathematical giftedness also seems to correlate with numerous factors—(see Appendix A for which factors were found in each study). These factors roughly fall into social, motivational, and cognitive domains.

Specifically, in the social and motivational domains, motivation, high drive, and interest to learn mathematics, practice time, lack of involvement in social interpersonal, or religious issues, authoritarian attitudes, and high socio-economic status have all been related to high levels of mathematical achievement. Speculatively, it is interesting to ask whether some of these factors may be related to the so-called Spontaneous Focusing on Numerosity (SFON) concept which appears early in life and means that some children have a high tendency to pay attention to numerical information (Hannula and Lehtinen, 2005). To clarify this question, longitudinal studies could investigate whether high SFON at an early age is associated with high levels of mathematical expertise in later life. Better assessment of individual variability is also important, for example, Albert Einstein (who was a gifted even if sometimes “lazy” mathematician; see e.g., Isaacson, 2008) was famously anti-authoritarian.

In terms of cognitive variables, we found that spatial processing, working memory, motivation/practice time, reasoning, general IQ, speed of information processing, short-term memory, efficient switching from working memory to episodic memory, pattern recognition, inhibition, fluid intelligence, associative memory, and motor functions were all associated with mathematical giftedness. As a caveat it is important to point out that mere “significance counting” (i.e., just considering studies with statistical significant results regarding a concept) can be very misleading especially in the typically underpowered context of psychology and neuro-imaging research (see e.g., Szucs and Ioannidis, 2017). However, considering the patchy research, this is the best we can do at the moment. In addition, even if meta-analyses were possible, these also typically only take into account published research, so they usually (highly) overestimate effect sizes especially from small scale studies (see Szucs and Ioannidis, 2017).

All in all, we found 9 of the 40 studies reporting positive correlations between WM performance and mathematical performance, five of which also had a strong spatial or visuo-spatial component in WM tasks. Of these, the following subcategories of WM were correlated with mathematical giftedness: visuo-spatial WM (3 studies), visual WM (2 studies), general WM (2 studies), spatial WM (1 study), and central executive WM (1 study). We also found 13 studies correlating high spatial processing ability with mathematical giftedness, of which one also had a short-term memory component. Of these, seven studies reported a correlation between visuo-spatial processing and mathematical giftedness, and six studies reported a correlation between spatial processing and mathematical giftedness (none of these 13 studies measured visual or visuo-spatial WM, even though it seems likely that there is a relationship between spatial/visuo-spatial processing abilities in general and spatial/visuo-spatial WM). In contrast with spatial abilities and WM, most other factors had many fewer studies supporting them, the next highest being high motivation/practice with four studies (see Table 1 in Appendix A of Supplementary Material).

It is worth noting that studies of math prodigies could not identify any special cognitive advantage of individuals with extreme performance in some areas of mathematics. Rather, these studies emphasized the role of very intensive, sustained practice. First, this may mean that the skills of those prodigies who were tested really mostly reflect long-term memory encoding through practice. Second, it important to consider that many prodigies are usually not professional mathematicians but show excellence in relatively mundane areas of mathematics, like fast calculation and remembering a large number of mathematical results (note: no prodigies in the studies reviewed above were professional or degreed mathematicians). Hence, the skills of prodigies may actually have not much to do with higher levels of mathematics performance and therefore they may not represent a good model of the cognitive structure of “real” mathematicians. With regard to this it is notable that some studies with degree-level mathematicians (e.g., Sella et al., 2016) found especially strong visuo-spatial skills in these populations, while this was not reported in case of prodigies.

Overall, it seems that visuo-spatial (memory) processes may play a key role in high level mathematical understanding. This conclusion would be similar to conclusions from studies demonstrating the importance of visuo-spatial memory for mathematical development in children and that its weakness is associated with selective impairment of mathematical function in children (e.g., Passolunghi and Siegel, 2001, 2004; Passolunghi and Mammarella, 2010; Szűcs et al., 2013, 2014; Mammarella et al., 2015; Szűcs, 2016).

If the importance of visuo-spatial working memory is confirmed across a very wide developmental landscape (from weak math achieving children through gifted children to adult mathematicians) that would provide very strong evidence for the central importance of visuo-spatial memory for mathematical function and understanding, perhaps through providing a mental workspace for mathematical operations (Szűcs et al., 2014).

### The literature suffers from low power and high false report probability

**Figure 1:**
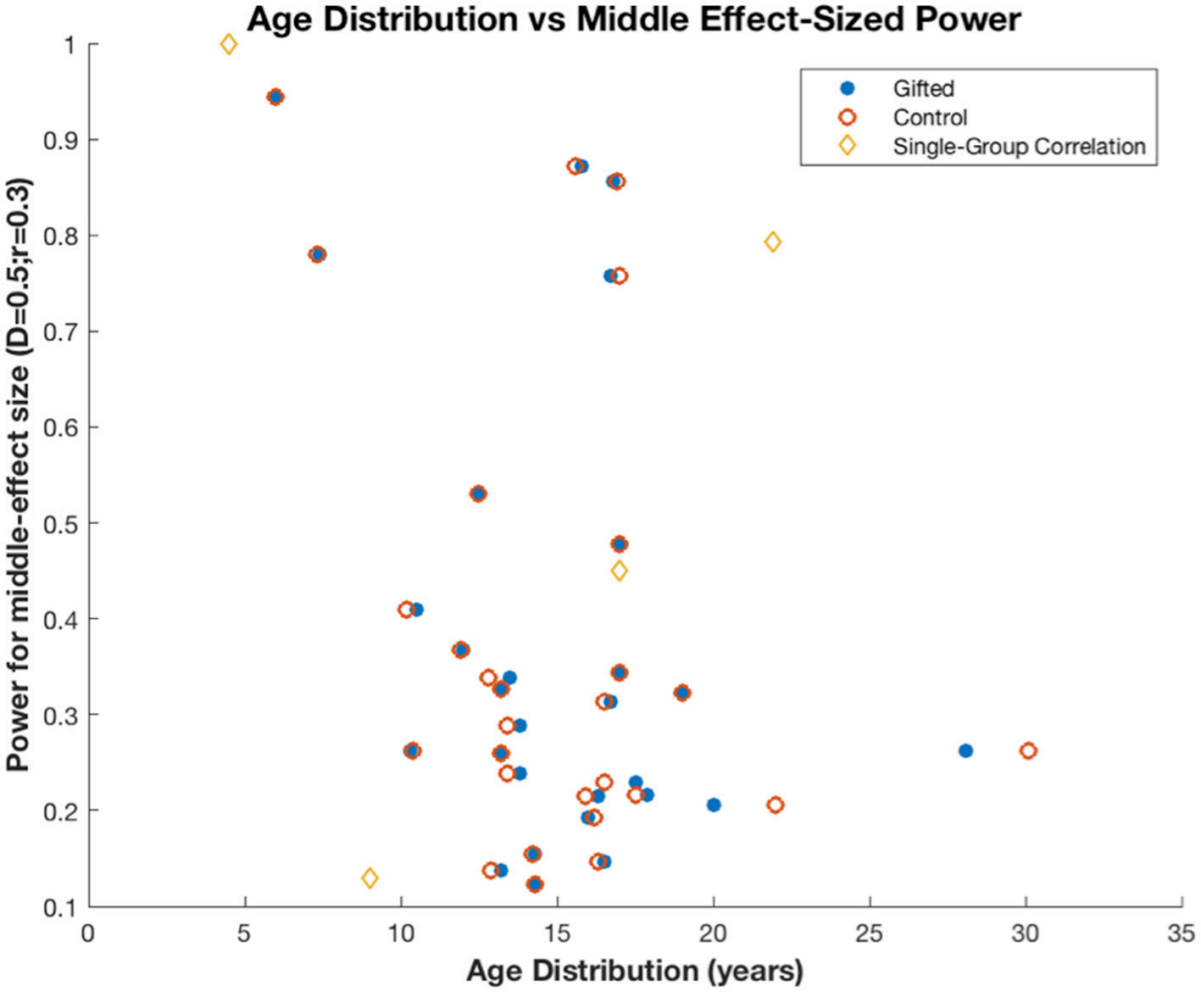
Power to detect medium sized effects in studies with various age groups (D = 0.5 for studies with math gifted and control groups; *r* = 0.3 for studies finding correlations with one group). Power does not change with age group. Power was relatively high in only eight out of 28 studies and was outstandingly high in a study with very large sample size (Robinson et al., 1996; *N* = 310).

The most apparent shortcoming of the math gifted literature is that most of the studies have relatively low participant numbers and hence have low power. Table 2 shows the power necessary to detect small, medium and large effect sizes (Sedlmeier and Gigerenzer, 1989). Out of 33 studies measured (note that studies with only 1 math gifted participant are not included), only the following 8 had power >0.5 to show medium sized effects (power range for all studies: 0.13–0.99): Barner et al. (2016), Benbow and Minor (1990), Kennedy and Walsh (1965), Leikin et al. (2013), Paz-Baruch et al. (2014), Robinson et al. (1996), Swanson (2006), and Wei et al. (2012). The studies in general also had very low power to show small effects (power range: 0.06–0.42). Figure 1 also demonstrates that power was similarly low across each age group studied. The above is important to consider because studies with low power have the triple-problem of missing true effects (false negatives), exaggerating measured effect sizes associated with statistically significant effects, and having a high false report probability (i.e., the probability that a statistically significant finding is false; Pollard and Richardson, 1987; Button et al., 2013; Szucs and Ioannidis, 2017). This highlights the need for exercising great caution when interpreting the results of these underpowered studies and also demonstrates the need for a larger number of studies measuring more variables with larger populations.

**Table 2:** The power of studies comparing mathematical gifted and control individuals to detect small (*D* = 0.3 or *r* = 0.1), medium (*D* = 0.5 or *r* = 0.3), and large (*D* = 0.8; or *r* = 0.5) effects.

**References**	**Participants**	**Variables measured**	**Groups (age in years)**	***D* = 0.2**	***D* = 0.5**	***D* = 0.8**
				***r* = 0.1**	***r* = 0.3**	***r* = 0.5**
Alexander et al., 1996	Adolescents	Brain Resting State	Gifted(*n.r*.): 30 Control(*n.r*.): 30	0.119	0.478	0.861
Amalric and Dehaene, 2016	Adult (academics)	Math, Verbal Processing	Gifted(28.1): 15 Control(30.1): 15	0.083	0.262	0.562
Barner et al., 2016	Children	General IQ, Math Ability, Non-verbal Reasoning, Visuo-spatial Processing, STM, WM	Gifted(*n.r*.): 100 Control(*n.r*.): 104	0.295	0.944	0.999
Benbow and Minor, 1990	Children (prodigious in math or reading or both; here only included math & reading)	Spatial Processing, Non-verbal Reasoning, Mechanical Comprehension, Vocabulary, General Knowledge	Gifted(*n.r*.): 106 Control(*n.r*.): 20	0.129	0.530	0.903
Dark and Benbow, 1990	Adolescents & College age	Math Ability, WM, Verbal Ability	Gifted(13.5): 20 Control(12.8): 20	0.095	0.338	0.693
Dark and Benbow, 1991	Adolescents	WM	Gifted(*n.r*.): 22 Control(*n.r*.): 19	0.096	0.344	0.702
Dark and Benbow, 1994	Adolescents	General Cognitive Skills	Gifted(*n.r*.): 11^*^ Control(*n.r*.): 10^*^	0.072	0.193	0.412
Desco et al., 2011	Adolescents	Math Reasoning, Non-verbal Reasoning	Gifted(13.8): 13 Control(13.4): 14	0.079	0.239	0.515
Hoppe et al., 2012	Adolescents	Visuo-spatial Processing	Gifted(16.7): 17 Control(16.5): 20	0.091	0.314	0.655
Hu et al., 2011	Children	WM, General IQ	Gifted(10.5): 25 Control(10.2): 25	0.107	0.410	0.792
Kennedy and Walsh, 1965	High school students	Cognitive IQ, Personality	Gifted(*n.r*.): 90 Control(*n.r*.): 63	0.227	0.856	0.998
Krause and Heinrich, 2003	Adolescents	Math Ability	Gifted(17.9): 12 Control(17.5): 12	0.076	0.216	0.466
Leikin et al., 2013	High school students	Non-verbal Reasoning, Math Ability, WM	Gifted (*n.r*.): 70 Control (*n.r*.): 87	0.236	0.872	0.999
Leikin et al., 2014	Adolescents	Non-verbal Reasoning, Math Ability, WM	Gifted(17.5): 7 Control(16.5): 49	0.078	0.229	0.494
Morsanyi et al., 2013	Children	Reasoning, Math Ability, Math Reasoning, Verbal Ability, Verbal Reasoning, Non-verbal Reasoning, General IQ	Gifted(10.3): 14 Control(10.4): 16	0.083	0.262	0.560
Navas-Sánchez et al., 2013	Adolescents	Brain Resting State	Gifted(13.8): 13 Control(13.4): 23	0.087	0.288	0.610
Navas-Sánchez et al., 2016	Adolescents	Brain Resting State	Gifted(13.2): 13 Control(13.2): 17	0.082	0.259	0.554
O'Boyle et al., 2005	Adolescents	Visuo-spatial Processing	Gifted(14.3): 6 Control(matching): 6	0.061	0.123	0.241
O'Boyle et al., 1991	Adolescents	Emotional Processing Verbal Abilities	Gifted(13.2): 6 Control(12.9): 8	0.063	0.137	0.276
O'Boyle et al., 1994	Adolescents	Motor, Verbal, Spatial Ability	Gifted(13.2): 24 Control(matching): 16	0.093	0.327	0.676
Paz-Baruch et al., 2014	High school students	Math Ability	Gifted(16.7): 41 Control(17): 96	0.186	0.758	0.989
Prescott et al., 2010	Adolescents	Spatial Processing	Gifted(14.3): 8 Control(14.2): 8	0.066	0.154	0.320
Robinson et al., 1996	Preschoolers and Kindergartners	Quantitative, Verbal, Visuo-spatial Processing	Subjects(*n.r*.): 310	0.422	0.999	1.000
Ruthsatz et al., 2014	Child Prodigies	General IQ, WM	Subjects(13): 9	0.058	0.130	0.322
Sella et al., 2016	Adults (Ph.D and post-docs)	Math Ability, Verbal and, Non-verbal Reasoning	Gifted(25.7): 19 Control(26.3): 19	0.092	0.323	0.670
Swanson, 2006	Children	Math and Reading Ability, Non-verbal Reasoning, WM, Visuo-spatial Processing, General Cognitive Ability	Gifted(7.4): 50 Control(7.3): 77	0.194	0.780	0.992
Tanaka et al., 2002	Adults (abacus experts and normal)	Math Ability	Gifted(20): 10 Controls(22): 13	0.074	0.206	0.442
Van Garderen, 2016	Children	General IQ, Math and Visuo-spatial Ability	Gifted(12.0): 22 Control(11.9): 22	0.099	0.367	0.736
Wei et al., 2012	Adults (college)	Basic Numerical Processing, Complex Numerical Processing, Spatial Processing, Language and General Cognitive Ability	Subjects(21.9): 80	0.144	0.793	0.999
Wu, 1996	Adolescents (Math Olympians)	Family Environment	Subjects(*n.r*.): 36	0.090	0.450	0.920
Zhang et al., 2014	Adolescents	Reasoning Ability	Gifted(16.3): 11 Control(15.9) 13	0.075	0.215	0.463
Zhang et al., 2015b	Adolescents	Verbal Reasoning	Gifted(16.3): 11 Control(15.9): 13	0.075	0.215	0.463
Zhang et al., 2015a	Adolescents	Math Ability	Gifted(16.5): 8 Control(16.3): 7	0.065	0.146	0.299

*Power was computed for two-tailed independent sample t-tests with α = 0.05 taking the ratio of the sample size of the to-be-compared groups into account, or Pearson correlations for studies with a single group investigating correlations (α = 0.05). Power was computed using the Matlab “sampsizepwr” function. n.r. = age means not reported. Studies which only had 1 participant to represent the math gifted were excluded*.

* Exact number for each group not reported

Importantly, low power may also explain the variable findings across studies (see section Heterogeneous Populations). Underpowered studies can produce highly diverse results with regard to single variables measured (Schmidt, 1992). Thus, we suggest that highly powered studies measuring numerous variables would converge on demonstrating the critical role of a few core variables for mathematical giftedness.

### Frequent use of invalid backward inference from neuroscience data

Another shortcoming of the literature reviewed here is that most of the neuroimaging studies tended to use “backward inference,” that is, they assumed the presence of certain cognitive activities based on neuroimaging data alone with no supporting behavioral information from the actual imaging paradigm. Such inference is likely to be invalid. This is because the literature links each brain region to a large number of cognitive functions [e.g., the intraparietal sulcus (IPS) has been connected to numerical processes, working memory, attention, and many other processes; see e.g., Szűcs et al., 2013; Cortex for review]. Consequently, the activation of a certain brain region on its own cannot be taken as evidence for the activation of a single cognitive function associated with it (Poldrack, 2006). It is thus concluded here that well-powered neuroimaging studies with clearly designed parametric functional manipulations, using appropriate supporting behavioral paradigms, are also lacking in the field. The current reverse “inferences” are invalid, and the truth of their conclusions remains undetermined.

### Importance of establishing math domain-specificity

Minimally, math domain-specificity must be established in order to draw clear conclusions. In other words, if brain activation is only shown in mathematics tasks, it cannot be then concluded that the brain activation is specific to math rather than to domain general functions. Additionally, if the task (operation) is available in other tests beyond math, then stimulus specificity should also be established, if possible. Not all studies reporting a cognitive or neural activation correlating with a mathematics task tested for math-domain specificity. Those which did, excluding multiple-factor correlation studies, are the following: Amalric and Dehaene (2016), Dark and Benbow (1990), Desco et al. (2011), and Sella et al. (2016).

### Causality

It is of high importance to note that causal claims require very strong evidence. For example, if a study finds that number-line knowledge is very refined in gifted mathematicians, it cannot then be concluded that good knowledge of number line leads to excellent mathematical performance. A high level of number line performance may simply be the consequence of sustained mathematical practice. Single time point measurements do not allow for determining causality; such studies can only point to the existence of a relationship between some variables. Studies reviewed here which suggest a causal relationship for math giftedness are the following: Amalric and Dehaene (2016), Fehr et al. (2011), Fehr et al. (2010), Jensen (1990), Minati and Sigala (2013), Navas-Sánchez et al. (2013), O'Boyle et al. (1991), O'Boyle et al. (2005), and Tanaka et al. (2002).

### The nature of links to mathematics

Another observation to note is that, while several cognitive factors have been proposed to correlate with mathematical giftedness, these processes (especially domain-general processes such as visuo-spatial and executive functioning) have primarily been studied in a general manner, to determine if correlations exist. To further advance the field, we now need more nuanced studies detailing the specifics concerning what unique contribution each of these processes may contribute toward mathematical giftedness.

### Interrelationships

Finally, the relationships between various factors found to correlate with math giftedness should be explored in a cohesive manner. For example, studies to date have focused almost exclusively on domain-general functions, domain-specific functions, or environmental influences/emotions. More large multi-factor research is needed investigating all of these together so that we can learn which are necessary as well as how they all cooperate toward a system of high mathematical performance. As a specific example, cognitive factors such as spatial processing and working memory should be explored alongside consideration of emotional factors such as a high drive to succeed and a strong work ethic. In this way, the relationship between these factors can be understood more accurately.

## Author contributions

DS conceived of and supervised this review. TM and DS evaluated the studies for fit. TM conducted the literature review, analyzed the articles, and wrote the initial draft. TM, DS, and EC revised drafts and edited the final manuscript.

### Conflict of interest statement

The authors declare that the research was conducted in the absence of any commercial or financial relationships that could be construed as a potential conflict of interest.
