# Supplementary material for: Cognitive and Neural Correlates of Mathematical Giftedness in Adults and Children: A Review
Source: Front Psychol. 2017 Oct 25;8:1646. doi: 10.3389/fpsyg.2017.01646 (PMC5661150; doi:10.3389/fpsyg.2017.01646)
Supplement: Supplementary file 1 [file Table1.docx]

**Appendix A**

| Factors | Sub-factors | # of studies | Studies |
| --- | --- | --- | --- |
| Spatial Processing | Visuo-spatial | 7 | Robinson et al. (1996); Van Garderen (2016); Desco et al. (2011); Hu et al. (2011); Sella et al. (2016); Ruthsatz et al. (2014); Amalric & Dehaene (2016) |
|  | Spatial | 6 | Benbow & Minor (1990); Hoppe et al. (2012); Lubinski & Benbow (2006); O’Boyle et al. (2005); Wai, Lubinski, & Benbow (2009); Wei et al. (2012) |
| Working Memory | Visuo-spatial | 3 | Leikin et al. (2013); Leikin et al. (2014); Tanaka et al. (2002) |
|  | Visual | 2 | Dark & Benbow (1991); Dark & Benbow (1994) |
|  | General | 2 | Ruthsatz (2014); Zhang, Gan, & Wang (2015) |
|  | Spatial | 1 | Barner et al. (2016) |
|  | Central Exec. | 1 | Swanson (2006) |
| Motivation/practice time |  | 4 | Kenney & Walsh (1965); Jenson (1990); Fehr et al. (2010); Fehr et al. (2011) |
| Reasoning | General | 1 | Zhang et al. (2015); |
|  | Independent of beliefs | 1 | Morsanyi et al. (2013) |
|  | Non-verbal | 1 | Benbow & Minor (1990) |
| General I.Q. |  | 2 | Kenney & Walsh (1965); Ruthsatz et al. (2014) |
| Speed of info. process. |  | 2 | Paz-Baruch et al. (2014); Swanson (2006) |
| Short-term memory |  | 1 | Dark & Benbow (1990) |
| Long-term mem. access |  | 1 | Minati & Sigala (2013) |
| Efficient switching from wm to episodic |  | 1 | Pesenti et al. (2001) |
| Pattern recognition |  | 1 | Leikin et al. (2014) |
| Inhibition |  | 1 | Swanson (2006) |
| Authoritarian attitudes |  | 1 | Kenney & Walsh (1965) |
| Lack of involvement in social, interpersonal, or religious issues |  | 1 | Kenney & Walsh (1965) |
| Socio-economic status |  | 1 | Wu (1996) |
| Fluid intelligence |  | 1 | Prescott et al. (2010) |
| Associative memory |  | 1 | Benbow & Minor (1990) |
| Attentional/strategic resources |  | 1 | Minati & Sigala (2013) |
| Motor functions |  | 1 | Hu et al. (2011) |
| Entropy reduction |  | 1 | Krause, Seidel, & Heinrich (2003) |

**Supplementary Table 1.** The table lists all cognitive factors reported to have statistically significant correlations with mathematical giftedness. Note that simply counting studies with statistically significant findings is not optimal and likely to be misleading. However, currently there are only very few available studies and they also measured very different variables. This state of the literature does not allow us to carry out a more principled effect size analysis.

**Appendix B**

|  |  |  | *Number of Hits* |  |  |
| --- | --- | --- | --- | --- | --- |
| *Search Terms* | *Google Scholar* | *Elsevier* | *PubMed* | *Scopus* | *Web of Science* |
| Math expertise | 244,000 | 6,830 | 285 | 153 | 112 |
| Mathematics expertise | 547,000 | 15,139 | 263 | 722 | 388 |
| Mathematical expertise | 550,000 | 29,245 | 426 | 2,083 | 574 |
| Math experts | 305,000 | 29,245 | 410 | 298 | 176 |
| Mathematics experts | 572,000 | 38,044 | 358 | 3,032 | 796 |
| Mathematical experts | 666,000 | 85,054 | 618 | 14,537 | 3,051 |
| Math proficiency | 119,000 | 2,909 | 229 | 254 | 166 |
| Mathematics proficiency | 210,000 | 4,589 | 212 | 633 | 382 |
| Mathematical proficiency | 126,000 | 4,781 | 257 | 523 | 225 |
| Math precocity | 7,880 | 87 | 8 | 4 | 3 |
| Mathematics precocity | 6,880 | 148 | 8 | 9 | 2 |
| Mathematical precocity | 10,700 | 282 | 10 | 37 | 24 |
| Math precocious | 13,700 | 462 | 45 | 21 | 25 |
| Mathematics precocious | 26,400 | 553 | 41 | 42 | 34 |
| Mathematical precocious | 21,600 | 1,173 | 60 | 76 | 46 |
| Math talented | 81,200 | 1,250 | 28 | 98 | 57 |
| Mathematics talented | 144,000 | 2,394 | 25 | 249 | 138 |
| Mathematical talented | 93,400 | 2,247 | 30 | 124 | 70 |
| Math gifted | 103,000 | 3,892 | 93 | 166 | 116 |
| Mathematics gifted | 201,000 | 6,590 | 78 | 401 | 259 |
| Mathematical gifted | 118,000 | 10,247 | 97 | 205 | 176 |
| Math giftedness | 12,400 | 227 | 22 | 29 | 25 |
| Mathematics giftedness | 16,500 | 339 | 15 | 74 | 59 |
| Mathematical giftedness | 11,600 | 296 | 22 | 80 | 75 |
| Math prodigy | 8,400 | 158 | 33 | 5 | 6 |
| Mathematics prodigy | 15,200 | 379 | 31 | 15 | 2 |
| Mathematical prodigy | 14,000 | 558 | 38 | 31 | 15 |
| Math prodigious | 11,700 | 286 | 7 | 2 | 1 |
| Mathematics prodigious | 30,000 | 651 | 7 | 16 | 4 |
| Mathematical prodigious | 29,400 | 1,044 | 9 | 34 | 11 |
| Math prodigiousness | 17,100 | 1,044 | 0 | 1 | 1 |
| Mathematics prodigiousness | 17,700 | 8 | 0 | 0 | 0 |
| Mathematical prodigiousness | 17,300 | 6 | 0 | 0 | 1 |

**Supplementary Table 2.** Search terms used for literature review and ‘hits’ per database.
